# On the effect of lateral stretch on the deformation energetics of biological membranes and the lipid dynamics within

**DOI:** 10.64898/2026.03.06.710217

**Published:** 2026-03-09

**Authors:** Yein Christina Park, Giacomo Fiorin, José D. Faraldo-Gómez

**Affiliations:** 1 Theoretical Molecular Biophysics Laboratory, National Heart, Lung and Blood Institute, National Institutes of Health, Bethesda, MD, United States; 2 Section on Sensory Physiology and Biophysics, National Institute on Deafness and other Communication Disorders, National Institutes of Health, Bethesda, MD, United States; 3 Graduate Program in Cell, Molecular, Developmental Biology and Biophysics, Johns Hopkins University, Baltimore, MD, United States; 4 Computational Structural Biology Section, National Institute for Neurological Disorders and Stroke, National Institutes of Health, Bethesda, MD, United States

## Abstract

A broad range of cellular functions involve transient or persistent changes in the morphology of lipid membranes, from the organellar to the molecular scale. By and large, the thermodynamics of these remodeling processes remain to be understood. Molecular Dynamics simulations enhanced by advanced sampling methods are uniquely suited to examine and quantitate these phenomena. Here, we focus on the cellular process known as mechanosensation and use the Multi-Map simulation method to quantify how applied lateral tension impacts the energetics of both global and localized membrane perturbations induced extrinsically. We also examine how tension impacts the dynamics of lipid molecules. We find that the conformational energetics of the membrane clearly differs when it is stretched, and that this difference increases with the magnitude of the applied tension. The reason is not that tension alters the mechanical properties of the lipid bilayer, such as its bending modulus, but rather that it opposes any reduction in the projected area of the membrane relative to that at rest, while the opposite is favored. It follows that tension may shift a conformational equilibrium of a protein that deforms the membrane differently in alternative functional states, if that difference also entails a change in the projected membrane area. Conversely, we find that stretch has little to no effect on the dynamics of lipids at the single-molecule level, implying it would also have no bearing on the lifetime of specific protein-lipid interactions. Finally, we show how changes in lipid composition that result in global membrane thinning can mimic the effect of lateral stretch without any applied tension.

## Introduction

Membrane protein function and regulation are deeply connected to the composition, morphology, and plasticity of the lipid bilayer. Indeed, it is increasingly apparent, based on computer-simulation studies and experimental high-resolution structural data, that the shape and chemical composition of the membrane in the vicinity of proteins are just as diverse as the folds of the proteins themselves. Localized shape deformations, for example, develop to prevent the exposure of polar amino-acids to the membrane interior, and to shield hydrophobic amino acids from water (1–4). These differentials in solvation energetics are remarkably large in magnitude (5,6), but to deform the lipid bilayer is also inherently costly. Therefore, the morphology of the protein-lipid interface ultimately reflects a trade-off between opposing energetic gains and penalties. The sensitivity of this balance, in turn, provides the cell with a means to regulate membrane protein function through mechanical inputs and variations in lipid content. Examples include diverse processes such as ion channel gating (7–11) or protein topology (12) and oligomerization (1,13) even for proteins whose biological function is seemingly unrelated to membrane morphology.

Despite the wide range of processes that are potentially affected or regulated by the shape or composition of the membrane, limited progress has been made to quantify the energetics of lipid bilayer deformations or their impact on lipid partitioning, particularly in ways that mimic the effects of membrane-embedded proteins. Computational methods based on continuum-mechanics theory have long been used to examine the determinants of membrane curvature (14,15) or thickness (16,17). The appeal of these methods is that they are conceptually elegant and do not require a large computing effort. They also rely on empirical descriptors of the membrane mechanical properties, such as the bending and compressibility moduli, that can be experimentally inferred for model cases. For many cases of interest, however, the necessary descriptors are often not measurable or verifiable. For example, to our knowledge it has not yet been determined whether a membrane subject to significant lateral stretch would retain the mechanical properties of an equivalent membrane in a tensionless state; similarly, how lipid-composition heterogeneity influences the mechanical properties of a membrane remains to be systematically examined. More broadly, heterogeneous lipid bilayers are challenging to model at the continuum-theory level because membrane shape and the spatial distribution of lipids are interdependent (18); no general analytical theory that we are aware of can anticipate with accuracy how different lipid species will distribute around a protein.

Computer simulations with molecular-scale resolution such as Molecular Dynamics (MD) have their own shortcomings, but as computing capacity and capability continue to increase, there is little doubt that simulations will remain the theoretical method of choice to examine the complex interplay between proteins and their surrounding membrane (19–21). The principal challenge that MD simulation studies face is the limited timescale of the processes that may be reliably examined, especially as the dimensions of the system under study reach the biological scale. A range of methodologies have been developed to overcome this limitation, however, collectively referred to as enhanced-sampling techniques (22,23). These approaches are designed to induce or increase the probability of rare events of interest, during a simulation that would otherwise yield little or no insight. This kind of advanced simulations can therefore reveal mechanistic and thermodynamic information about processes that would be spontaneously observable only for longer time scales. Enhanced-sampling techniques have been used extensively in membrane biology, for example to study the substrate transport mechanisms (24,25), ion permeation (26), protein oligomerization (27,28), the formation of transmembrane pores (29,30), or local changes in membrane shape (31–36). Recently, we developed and validated an advanced MD simulation method of this kind, named Multi-Map, explicitly designed to characterize the energetics of membrane deformations of any shape or scale, for membranes of any lipid composition (37–40). Unlike continuum-mechanics models, the free-energy landscapes obtained through this approach reflect the complex structural dynamics and interatomic forces recorded in simulation, rather than pre-conceived theoretical assumptions.

Here, we seek to gain insights into an important cellular mechanism in sensory biology, known as mechanosensation. To date, several classes of mechanosensitive membrane proteins have been identified, including those that sense mechanical stimuli via tether to other proteins (41) and those that can respond directly to changes in the membrane environment (42). In simple terms, this latter group of mechanosensitive proteins are those whose functional state changes when the surrounding membrane is laterally tensioned. More specifically, we leverage both conventional and enhanced-sampling simulations, based on the Multi-Map method, to investigate how lateral stretch influences the elementary mechanical properties of the membrane, and to quantify the energetics of membrane deformations of the kind observed around mechanosensitive proteins. We also examine the effects of applied lateral stretch on lipid dynamics, asking whether it appreciably alters lipid mixing, diffusivity and spatial localization. Finally, we assess whether the influence of lateral stretch on the morphological energetics of a membrane can be reproduced in a tensionless state if the lipid composition of the membrane is altered.

## Methods

The MD simulations in this study were computed with either GROMACS 2018.8 (43) or NAMD 2.14 (44), as noted. In all cases, simulations were based on the MARTINI force field, version 2.2 (45). In-house code was developed to make the MARTINI 2.2 parameter and topology files compatible with NAMD, available at https://github.com/1004parky/martini2_in_namd.

### Construction and equilibration of molecular systems

To examine how the spontaneous shape fluctuations of a membrane at rest change under stretch, we used lipid bilayers of 200 or 5,000 POPC molecules constructed for a previous study (37), whose lateral dimensions are 78 × 78 Å^2^ and 390 × 390 Å^2^, respectively. To examine how tension influences the energetics of induced deformations, we generated two additional bilayers, featuring different lipid compositions. These bilayers were constructed using the *insane* protocol (46) and consist of 400 lipids per leaflet (800 lipids total, 155 × 155 Å^2^), solvated with 9:1 water:antifreeze-water (45). The compositions of these bilayers is either 100% POPC or 50% POPC and 50% DLPC, distributed symmetrically between the two leaflets. The number of water particles for the pure POPC system is 22,944 and for the POPC/DLPC system is 23,753.

The two newly constructed 800-lipid bilayers were energy-minimized with GROMACS using the steepest-descent algorithm, for 500,000 steps. To begin to equilibrate these systems, we carried out three sequential MD simulations of 1 ns each, also in GROMACS, increasing the integration timestep from 2 to 10 to 20 fs; the temperature was set at 300 K through velocity-rescaling (47) and the pressure was set at 1 atm with the Berendsen semi-isotropic barostat (48). A cut-off value of 12 Å was used for both electrostatics and van der Waals interactions. After the third step, a fourth equilibration was carried out with NAMD, for 100 ns, also at 300K and 1 atm, using a timestep of 25 fs and 12-Å cutoff for all nonbonded interactions. Unless otherwise noted, all the subsequent simulations used to evaluate the energetics and dynamics of the membrane in the microsecond timescale were calculated under the same conditions and energy functions.

### Simulation of applied lateral tension

Hereafter we use the terms “lateral stretch”, or alternatively “mechanical tension” (49) to refer to an outward stretching force applied to a lipid bilayer in the direction parallel to its midplane. This force is distinct from the persistent interfacial effect that exists at the lipid-water boundary, known as “surface tension” or “interfacial tension”. To stretch the membrane laterally in simulation, we set the target pressures along the membrane plane, ${P_{xx}}^{\text{target}}$ and ${P_{yy}}^{\text{target}}$, to a value that differs from that along the membrane perpendicular, ${P_{zz}}^{\text{target}}$. The applied tension γ relates these three target pressures according to the following relationship:

(1) $$\gamma =L_{z}\left(P_{zz}^{\mathrm{target}}-\frac{\left(P_{xx}^{\mathrm{target}}+P_{yy}^{\mathrm{target}}\right)}{2}\right)$$

where $L_{z}$ is the length of the simulation box along the membrane perpendicular, and ${P_{zz}}^{\text{target}}=1$ atm. Note also that the target pressures ${P_{xx}}^{\text{target}}$ and ${P_{yy}}^{\text{target}}$ are equal when the simulation system is isotropic on the XY plane, as is the case in this study. To enforce these target pressures, we used the Langevin-piston method built into NAMD, using a 2-ps period and 1-ps decay. The 200-lipid and 5,000-lipid bilayers were simulated both with and without tension, with several values of γ ranging from 0 to 10mN/m(1mN/m=1dyn/cm); the 800-lipid bilayers were simulated at tension values of 0 and 10mN/m.

### Definition of Multi-Map collective variables for different membrane perturbations

To induce different shape perturbations in the membrane and to calculate the associated free-energy profile in each case, we used the Multi-Map method (37) in combination with the umbrella-sampling technique (50). Specifically, we used two collective variables $\xi_{\text{upper}}$ and $\xi_{\text{lower}}$ to independently describe and define the morphology of each bilayer leaflet, through volumetric maps that represent their shape both at rest and at different magnitudes of a deformation of interest. For computational simplicity, only the lipid phosphate groups were considered in the definition of these collective variables; the variable used as reaction coordinate in the umbrella-sampling calculations, ξ, is the sum of $\xi_{\text{upper}}$ and $\xi_{\text{lower}}$.

To define an undeformed reference state of the membrane, we used volumetric maps that take the form of 1D Gaussian functions along the direction perpendicular to the membrane plane. Since both lipid composition and lateral tension influence the thickness of the bilayer at rest, the center of each Gaussian function was uniquely specified for each simulation system, based on analysis of the equilibration trajectory for that system (Figure S1). Because the phosphate groups naturally reside within the width of these Gaussian functions when the membrane is at rest, this design ensures that application of an umbrella potential on the Multi-Map variable does not deform the membrane, nor does it incur a free-energy cost, as is required for a valid reference state.

**Figure 1.**
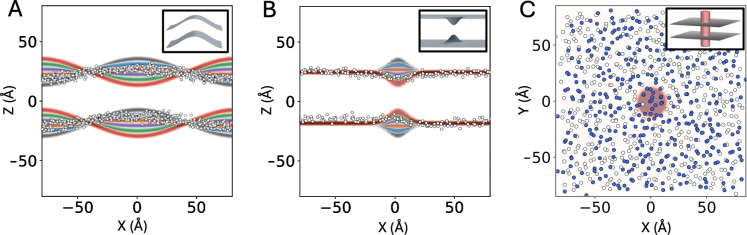
Volumetric maps used to induce and quantify perturbations in membrane shape and lipid distribution through Multi-Map simulations. (**A**) A cross-section of the set of maps used to study the energetics of a sinusoidal bending deformation in a POPC membrane. Overlaid is a cross-section of a snapshot of the phosphate layers, for $\xi_{\text{bend}}$ ≈ 10, with phosphate groups represented as white circles. The inset shows a 3D contour of one set of maps, for each of the bilayer leaflets. (**B**) A cross-section of the set of maps used to study the energetics of a localized thickness defect. Overlaid is a cross-section of a snapshot of the phosphate layers, for $\xi_{\text{thickness}}$ ≈ 0.7, represented as in (A). The inset shows a 3D contour of one set of maps. (**C**) A cross-section of the cylindrical map used to examine lipid dynamics. Overlaid is an XY-projection of a snapshot of the phosphate layers, for $\xi_{\text{occupancy}}$ ≈ 15. Lipids designated as ${POPC}_{\text{special}}$ are marked in blue. The inset shows a 3D contour of the target cylindrical volume, intersecting both leaflets of the bilayer.

To induce a global bending deformation of membrane and to quantify its energetics, a set of volumetric maps were generated by convoluting the reference-state 1D Gaussian functions with a series of 1D sinusoidal functions defined as:

(2) $$z_{bend}(x,y)=W_{bend}cos\left(\frac{2\pi x}{L}\right)$$

where L is the side-length of the membrane, and $W_{\text{bend}}$ is equal to −10,−6,−2,+2,+6, or +10Å (Figure 1A). As mentioned above, these 6 functions were used to define a Multi-Map variable for each leaflet, $\xi_{\text{upper}}$ and $\xi_{lower}$; their sum, $\xi_{bend}$, was then used as the umbrella-sampling coordinate.

To induce a localized thickness defect, the reference-state 1D Gaussian functions were convoluted with a series of 2D Gaussian curves of the form:

(3) $$z_{\text{thickness}}(x,y)=W_{\text{thickness}}e^{-0.5\left(x^{2}+y^{2}\right)/\sigma^{2}}$$

where $W_{\text{thickness}}$ is equal to −10,−6,−2,+2,+6 or +10Å, and σ determines the area affected by the thickness defect (Figure 1B). Specifically, we set σ=8Å such that the defect is about 40 Å in diameter. Analogously to the global deformation, these six maps were used to define a Multi-Map variable for each leaflet, $\xi_{\text{upper}}$ and $\xi_{\text{lower}}$, and these were combined into an overall reaction coordinate, $\xi_{\text{thickness}}$. When defining $\xi_{\text{upper}}$ and $\xi_{\text{lower}}$, however, maps with opposing sign of $W_{\text{thickness}}$ were considered for the upper leaflet and lower leaflet; therefore, negative values of $\xi_{\text{thickness}}$ correspond to membrane thickening (both leaflets bulging away from the midplane), while positive values reflect membrane thinning (both leaflets squeezing toward the midplane).

Finally, we used the Multi-Map method to induce the enrichment or depletion of a subset of the lipids in the membrane in a specific region, as a means to examine the impact of lateral stretch on lipid dynamics. Specifically, this subset of lipids, referred to as ${POPC}_{\text{special}}$, are 50% of the POPC molecules in each leaflet, selected as random. The region targeted is a cylindrical volume of radius *R* of 10 Å, whose axis perpendicular to the membrane, spanning beyond both leaflets (Figure 1C). To induce the enrichment or depletion of ${POPC}_{\text{special}}$ in this target region, we defined a Multi-Map variable $\xi_{\text{occupancy}}$ that quantifies the total occupancy of the cylindrical volume by those lipids. For a given simulation snapshot, we defined the occupancy for each lipid i in the ${POPC}_{\text{special}}$ subset as:

(4) $$o_{i}(x,y)=\left\{\begin{array}{ll} 1 & \mathrm{if}\;r_{i}<R\\ e^{-0.5r_{i}^{2}/\sigma^{2}} & \mathrm{if}\;r_{i}\geq R \end{array}\right.$$

where $r_{i}$ denotes the distance from lipid i to the center of the cylinder, projected on the membrane plane. $\xi_{\text{occupancy}}$ is therefore the sum of $o_{i}$ over all ${POPC}_{\text{special}}$ lipids. Note that that occupancy function in Eq. 4 decays smoothly for $r_{i}\geq R$, specifically as a Gaussian of width σ=2Å. This design ensures that $\xi_{\text{occupancy}}$ is continuous and differentiable. As with the other membrane perturbations described above, enhanced sampling simulations based on $\xi_{\text{occupancy}}$ enabled us not only to control the population of ${POPC}_{\text{special}}$ lipids in the target cylindrical volume, but also to quantify the associated free-energy gain or cost.

### Umbrella-sampling simulations using Multi-Map and interpretation of free-energy profiles

As mentioned, we used the umbrella-sampling method (50) to calculate the potential of mean force (PMF) along the reaction-coordinate defined by each of the Multi-Map variables ξ described above. To do so, we employed the Colvars module (51,52) interfaced with NAMD (44). In all cases, we initialized the umbrella-sampling windows with coordinates sequentially extracted from a 600-ns steered-MD trajectory in which a moving harmonic restraint was applied onto ξ, increasing gradually over 500 ns, with force constant of 5 kcal/mol. Once the initial coordinates were obtained, each window was simulated independently, with sampling times ranging from 1 to 7 μs per window; the first 300-ns fragment in each window was however considered equilibration time and excluded from the calculation of free energies. The weighted-histogram analysis method (WHAM) (53) was used to analyze the rest of the trajectories and derive the PMF as a function of the value of ξ in each case. For each of the PMF profiles discussed in this study we ascertained that the sampling times per window exceed the convergence time of the PMF (Figure S2).

The global bending deformation (Figure 1A) was induced by sampling values of $\xi_{\text{bend}}$ from 0 to 20, in a series of 41 windows spaced by 0.5. The localized thickness deformation (Figure 1B) was induced by sampling values of $\xi_{thickness}$ from −1.15 (thickening) to 1 (thinning), in a series of 44 windows spaced by 0.05. The enrichment or depletion of ${POPC}_{\text{special}}$ was induced by sampling values of $\xi_{\text{occupancy}}$ (Figure 1C) from 0 (depletion) to 16 (enrichment), in a series of 17 windows spaced by 1. The force constants of the static umbrella-potentials used in these calculations were 5 kcal/mol for $\xi_{bend}$ and $\xi_{\text{occupancy}}$ and 500 kcal/mol for $\xi_{thickness}$. Note that because the volumetric maps that underlie $\xi_{bend},\;\xi_{thickness}$, and $\xi_{\text{occupancy}}$ are defined in absolute Cartesian space, all umbrella-sampling simulations also included a harmonic restraint applied to the center of mass of the bilayer, only along the Z-coordinate (membrane perpendicular), to ensure that the lipid bilayer remains in the frame of reference where the maps were constructed. This additional restraint has no effect on the results of the calculations, as the energy function used in our simulation systems is invariant to translations of the reference frame.

By definition, the collective variables $\xi_{bend},\;\xi_{thickness}$, and $\xi_{\text{occupancy}}$ are continuous and derivable functions of the atomic coordinates, as is required for any component of the energy function used in an MD simulation. However, while $\xi_{\text{occupancy}}$ is an intuitive descriptor (it measures a number of molecules), $\xi_{bend}$ and $\xi_{thickness}$ are dimensionless quantities that might not be generally intuitive. Therefore, the free-energy profiles originally calculated using $\xi_{bend}$ or $\xi_{thickness}$ as reaction-coordinates are shown instead as functions of two physical descriptors that, although computed *a posteriori*, are linearly correlated with $\xi_{bend}$ and $\xi_{thickness}$ (Figure S3). These descriptors are the peak curvature of the membrane, c, in lieu of $\xi_{bend}$, and the hydrophobic thickness of the membrane at the center of the defect, t, in lieu of $\xi_{thickness}$.

To derive the peak curvature of the membrane for a given simulation snapshot, we first calculated the map of the total curvature c(x,y) that best fits the map of the membrane midplane, h(x,y). Because the deformation depends only on the x coordinate (Eq, 2), the following expression can be used:

(5) $$c\left(x,y\right)=\frac{-h_{xx}\left(x,y\right)}{{\left(1\;+\;{\left(h_{x}\left(x,y\right)\right)}^{2}\right)}^{3/2}}$$

where the x suffix indicates a derivative along that component. We defined the mean value of c in each umbrella-sampling window as the average of the 1% largest values in the corresponding c(x,y) map. A linear regression between the mean values of $\xi_{bend}$ and the mean values of c for each of the umbrella-sampling windows used in the calculation of PMF($\xi_{bend}$) produced an analytic relationship between $\xi_{bend}$ and c, which we used to translate PMF($\xi_{bend}$) into PMF(c) (Figure S3A).

To quantify the hydrophobic thickness of the bilayer defect in a given simulation, we evaluated the mean distance between the outer and inner ester-group layers in an area within a radius of 0.04 × (box length) from the center of the defect (Figure S4). We evaluated the bulk thickness $t_{b}$ analogously, but in an area outside a radius of 0.3 × (box length) from the center of the defect, and within a radius of 0.425 × (box length) from that center (Figure S4). To translate PMF($\xi_{thickness}$) into PMF(t), or $PMF\left(t-t_{b}\right)$, we again used the relationship between these variables deduced from a linear regression between the mean values of $\xi_{thickness}$ and the mean values of t for each of the umbrella-sampling windows used in the original calculation of PMF($\xi_{thickness}$) (Figure S3B, S3C).

### Evaluation of membrane structure and dynamics

The MOSAICS software suite (54) was used to analyze several descriptors of membrane structure and dynamics as observed in our simulations. Specifically, the *zcoord*, *midplane*, and *delta_plot* programs were used to calculate the membrane midplane and thicknesses based on the coordinates of the lipid ester groups (GL1/2). Lipid enrichment was evaluated with the *2d_enrichment* program. Each of these programs produced a 2D projection of the quantity analyzed across the plane of the membrane, for a designated simulation time-window. Lipid diffusion and kinetics were evaluated with the *lipid_mixing* program, which reports the fraction of lipids *j* that reside in the vicinity of lipid *i* (e.g. the first solation shell) for longer than a certain elapsed time (10 ns in our work). Fluctuation spectra were calculated with MDAnalysis (55) and fitted against theoretical predictions as summarized in the Results section; statistical uncertainties for fitted parameters were bootstrapped using 50,000 samples around the computed points (56).

## Results

### Effect of applied lateral stretch on membranes at rest

**Figure 2.**
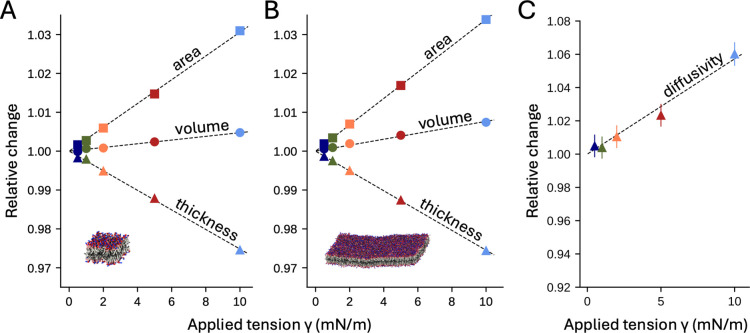
Effects of applied lateral stretch on membranes at rest. (**A**, **B**) Changes in area per lipid (squares), hydrophobic thickness (triangles), and volume (circles) caused by application of lateral tension of increasing magnitude to bilayers of 200 (A) or 5,000 (B) lipids. All values are in reference to the zero-tension condition. For each membrane and each tension condition, the values plotted derive from analysis of about 100 μs of sampling; the estimated statistical errors are smaller than the size of the symbols. (**C**) Changes in diffusivity D, for the 5,000-lipid bilayer, upon application of lateral tension of increasing magnitude, relative to the zero-tension condition. For each condition, D was deduced by calculating the linear slope of the relationship MSD = 2*D*τ, where MSD is the mean-squared displacement of all lipids in the membrane after an elapsed time τ in the 0 to 1 μs range; sampling errors were estimated from the spread of MSD values over lipid molecules.

We started by studying the effect of lateral tension on several key descriptors of membrane structure and dynamics, namely area per lipid molecule, volume, thickness, and lipid diffusivity. The basis for this analysis is a series of conventional MD simulations of both small and large POPC bilayers (200 and 5,000 lipids, respectively) with and without applied tension, approximately 100 μs in length for each value of γ (Eq. 1). We found that all the descriptors examined vary linearly with the magnitude of the applied tension (Figure 2A, 2B). Intuitively, lateral stretch causes the area per lipid to increase and the membrane thickness to decrease. In particular, the area per lipid increases at a relative rate of 0.31% per mN/m for the 200-lipid bilayer, and 0.34% per mN/m for the 5,000-lipid bilayer. (The inverse of these rates of change provides an estimate of the compressibility modulus $K_{A}$ for our POPC bilayers, ranging from 322 to 294 mN/m.) These changes in area per lipid outpace the changes in thickness, and so we observe an increase in membrane volume as the applied tension increases, and therefore a reduced lipid density. The increase in tension also results in an increase in lipid diffusivity, at a rate of ~0.6% per mN/m (Figure 2C). This change appears to reflect the abovementioned increase in the area per lipid: lipid diffusion is a Brownian process that requires adjacent lipid molecules to exchange their relative positions on the membrane plane. As the area per lipid increases, the cumulative displacements that result from these exchanges also become larger, and hence the value of the diffusion constant D is also increased (Figure 2C).

**Figure 3.**
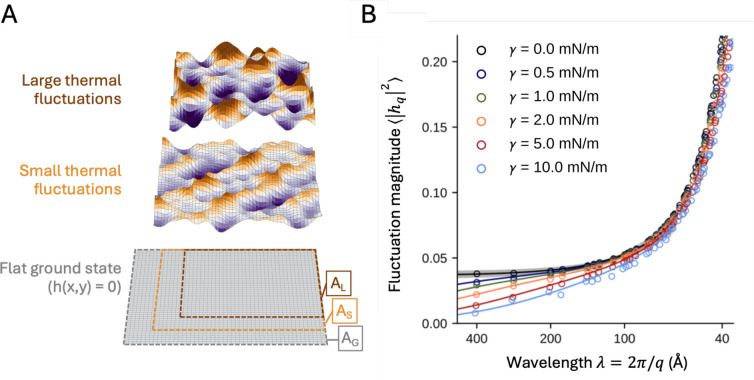
Impact of applied lateral tension on the spontaneous fluctuations of a bilayer at rest. (**A**) Illustrations of a membrane midplane in three different states, randomly generated in Python: a perfectly flat ground state, and two others with small and large thermal fluctuations, with color intensity (purple or orange) indicating larger amplitudes. Note how spontaneous thermal fluctuations naturally decrease the projected area of the membrane, from A_G_ (grey) to A_S_ (orange) to A_L_ (brown), respectively. Fluctuation modes such as these coexist at room temperature in the absence of stretch. However, upon application of lateral stretch, changes in projected area incur an energetic cost, given by γ (A_G_ – A_S_) or γ (A_G_ – A_L_) (Eq. 6). Fluctuations of larger magnitude and wavelength are thus increasingly unlikely to occur spontaneously under applied tension. (**B**) Fluctuation spectra for a 5,000-lipid bilayer, calculated under different lateral tensions, derived from conventional MD simulations of approximately 100 μs for each condition. The magnitudes of the observed fluctuation modes are plotted as circles. The black line corresponds to the best fit of Eq. 7 against the simulation data with γ = 0, which results in *k*_c_ = 16.3 ± 0.7 kcal/mol and *k*_t_ = 0.15 ± 0.02 kcal/mol/Å^2^. The gray area around the black solid line denotes the 95% confidence interval. Each of the other solid lines is Eq. 7 with the corresponding value of γ and the values of $k_{c}$ and $k_{t}$ obtained for γ = 0, with no additional fitting.

Next, we examined two important mechanical properties of the membrane, namely the bending modulus, $k_{c}$, and the tilt modulus, $k_{t}$. Both descriptors can be derived from analysis of a membrane at rest, or more specifically, from analysis of the spontaneous fluctuations of the membrane midplane. Following the Monge parameterization, the membrane midplane is defined as the function h(x,y), where h denotes a vertical displacement relative to a flat plane. The ground state for a symmetric bilayer with no preferred curvature therefore corresponds to h(x,y) = 0. Thermal fluctuations, however, will naturally cause deviations from this idealized state, even for a membrane at rest (Figure 3A). In this regime, the bending and tilt moduli contribute to define the likelihood of a given shape fluctuation, through the following expression of the energy of the membrane (57,58):

(6) $$G_{\gamma }[h,n]=\iint \left[\frac{1}{2}k_{c}(\nabla \cdot n{)}^{2}+\frac{1}{2}k_{t}|n-\nabla h{|}^{2}\right]dx\;dy+\gamma \Delta A$$

where n(x,y) is a position-dependent vector describing the local lipid orientation across the XY plane, ∇⋅n is the divergence of that vector, and ∇h is the gradient of the h(x,y) function. Note that the γΔA term in Eq. 6 is only relevant when the membrane is under stretch (59,60). Here, $\Delta A=A_{\text{ground}}-A$ quantifies the decrease in the area of the membrane (projected onto the XY plane) when the shape of the midplane fluctuates away from the perfectly flat ground state (Figure 3A). Note also that ΔA is positive by definition; under outward stretch, i.e. when γ>0, Eq. 6 predicts that fluctuations that reduce the projected area incur an energy penalty. Finally, note that while γ appears explicitly only in the last term of Eq. 6, to our knowledge it has not yet been determined whether $k_{c}$ and $k_{t}$ implicitly depend on it as well.

At a temperature T, the energy function in Eq. 6 predicts a spectrum of fluctuations of h whose magnitude is given by:

(7) $$\left.{\left.\langle |h_{q}\right|}^{2}\right\rangle =\frac{k_{B}T}{k_{c}q^{4}\;+\;\gamma q^{2}}+\frac{k_{B}T}{k_{t}q^{2}}$$

where q=2π/λ, and λ is the wavelength of the fluctuation. The first term of Eq. 7 reflects the fluctuation spectrum predicted by the Helfrich-Canham theory under lateral tension (49,61), while the second term accounts for the fluctuations of the lipid orientation vector (57,58). Thus, to examine whether the bending and tilt moduli are altered under stretch, for each value of γ we used our MD trajectories of the 5,000-lipid bilayer to compute ${\left|h_{q}\right|}^{2}$, following a methodology described previously (39) and derived the values of $k_{c}$ and $k_{t}$ that result in the best fit between the simulation data and Eq. 7.

We first considered the spectrum obtained for the unstretched bilayer, i.e. using γ=0, and were able to derive values for both moduli with very good statistical precision, namely $k_{c}=16.3\pm 0.7\;kcal/mol,\;k_{t}=0.15\pm 0.02\;kcal/mol/{\text{Å}}^{2}$ (Figure 3B). (Both values are in good agreement with previous studies of POPC bilayers under comparable conditions (62,63).) We then proceeded to examine the fluctuation spectra obtained under different values of the applied tension. Strikingly, we found that no significant changes in either parameter were required to fit Eq. 7 onto the simulation data for any of the other tension conditions, up to γ=10mN/m (Figure 3B).

The finding that both $k_{c}$ and $k_{t}$ are essentially unchanged when the membrane is under stretch has an important implication, namely that the area term in Eq. 6 is sufficient to capture the differential effect of applied lateral tension on the energetics of the membrane at rest. Indeed, our simulation data reveals the nature of this effect at the structural level. Specifically, we observe that lateral stretch suppresses the thermal fluctuations of the membrane that are larger in scale, particularly those of wavelength greater than 100 Å (Figure 3B). For example, 5 mN/m of applied tension suppresses the magnitude of the 400 Å-wavelength fluctuations by ~75%, while 100 Å-wavelength fluctuations are suppressed by ~10% (Figure 3B). As pointed out, this effect does not appear to reflect a change in either the bending or tilt moduli. The reason for this “flattening” of the bilayer is instead that large-scale fluctuations transiently reduce the area of the membrane, in projection, compared with the perfectly flat, ground state (Figure 3A). As predicted by Eq. 6, our simulations demonstrate that these transient compressions are increasingly penalized under stretch, and thus less likely to develop spontaneously.

### Effect of applied lateral stretch on global bending of the membrane

**Figure 4.**
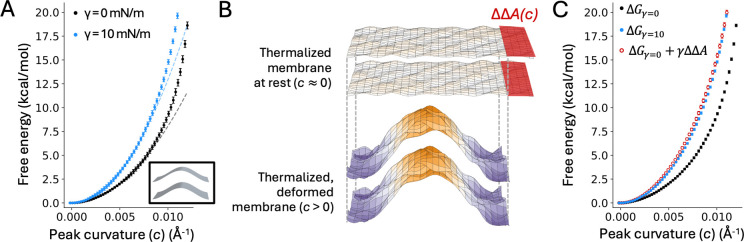
Effect of lateral tension on the energetics of membrane bending. (**A**) Potential of mean force (PMF) for a sinusoidal deformation of increasing curvature. The free energy is plotted as function of the peak curvature of the deformation, *c*, under no tension (black) or under applied lateral tension of 10 mN/m (blue). The PMF curves were calculated with the Multi-Map method in combination with umbrella sampling, using an 800-POPC lipid bilayer. Each umbrella-sampling window was 1 μs. Fragments of 200 ns were used to estimate average values of the PMF (circles) and standard deviations, σ (bars); the error bars in the plot (2σ) range from 0.01 to 0.39 kcal/mol. The dashed line is a quadratic fit (*y* = *ax*^2^) to the points below 5 kcal/mol (*a* = 80,545 kcal×Å^2^/mol for γ = 0 and 129,393 kcal×Å^2^/mol for γ = 10 mN/m). (**B**) Schematic illustrating the changes in projected area of the membrane as a result of the sinusoidal deformation, with color intensity (purple or orange) indicating larger amplitudes relative to the monolayer midplane. ΔΔA(*c*) is the difference in projected area between two thermalized states of the membrane, namely a reference, undeformed state with c≈0 and a deformed state with c>0. Note that the projected area of both states is smaller than that of a perfectly flat, idealized ground state. (**C**) Same data as in (A) with an additional free-energy profile (red) obtained by adding γΔΔA(*c*) to the PMF(*c*) profile obtained with no applied tension (black). The errors in this additional profile, ranging from 0.002 to 0.26 kcal/mol, are the sum of the errors in the PMF calculated for γ=0 and the errors in ∆∆A calculated with Eq. 8.

Next, we sought to examine how lateral tension influences the energetics of membrane bending on a larger magnitude, beyond what is attainable through thermal fluctuations. To do so, we used the Multi-Map simulation method (37) to induce a sinusoidal deformation of varying curvature (Figure 1A) and to quantify the associated free-energy change, with and without applied tension. These calculations are based on a lipid bilayer of smaller side-length (L≈155Å) than that considered in the previous section (L≈390Å), to examine the effect of tension at length scales comparable to the size of typical membrane proteins. The resulting profiles of the free energy as a function of the induced peak curvature, also known as potentials of mean force (PMF), are shown in Figure 4. Consistent with our previous studies (38–40) we observe that the energetic cost of bending the membrane increases with the magnitude of the induced curvature (Figure 4A). This free-energy cost is well-described by a quadratic function at lower curvature values, as would be expected from Eq. 6. However, as the curvature of the deformation increases, the free-energy profiles deviate significantly from that prediction (Figure 4A), underscoring why simulationbased free-energy calculations are required to quantify strongly deformed states.

Under applied lateral tension, we find that the cost of membrane bending is substantially greater (Figure 4A): for example, imposing a $0.01\;{\text{Å}}^{-1}$ curvature becomes ∼5.5kcal/mol more costly if γ=10mN/m. As shown in the previous section, this increased stiffness is not due to a change in the value of the bending or tilt moduli under stretch. Instead, we hypothesized that this change in the bending energetics might be related to the change in the XY-projected area caused by the deformation of the membrane, as noted for the spontaneous fluctuations at rest (Figure 4B). To examine this hypothesis, we calculated the relative change in projected area, ΔΔA, associated with a sinusoidal deformation of the form given by Eq. 2. Specifically, for a sinusoidal deformation $\Delta \Delta A=0.25c^{2}q^{-2}L^{2}$, where c is the peak curvature, q=2π/L and L is the side-length of the membrane. The simplified form for the area change is:

(8) $$\Delta \Delta A\left(c\right)=\frac{L^{4}}{16\pi^{2}}c^{2}\;$$

To derive ΔΔA from Eq. 8, we calculated the mean values of the peak curvature (c) and unit-cell length (L) in each of the umbrella-sampling simulations used to calculate the PMF profiles discussed above. Then, we added a term equal to γΔΔA, with γ=10mN/m, to the PMF derived for the membrane without tension. As shown in Figure 4C, the resulting PMF is nearly identical to that directly calculated for the membrane under stretch. Even more strikingly, Eq. 8 reproduces the difference between the two PMFs well past the point where the PMF curves cease to be quadratic in c. This finding implies that for this type of membrane deformation, the increased stiffness of the membrane under lateral stretch is explained almost entirely by the degree of compression of its projected area.

### Effect of applied lateral stretch on localized thinning and thickening of the membrane

We next examined the effects of lateral tension on the energetics of a localized change in membrane thickness, of the kind often observed at the interface between lipids and proteins. To do so, we used the Multi-Map method to create and characterize Gaussian-shaped deformations of increasing magnitude, defined to be symmetric relative to the bilayer midplane (Figure 1B). That is, through this approach we were able to directly quantify the energetics of membrane thinning or thickening under different tension conditions.

**Figure 5.**
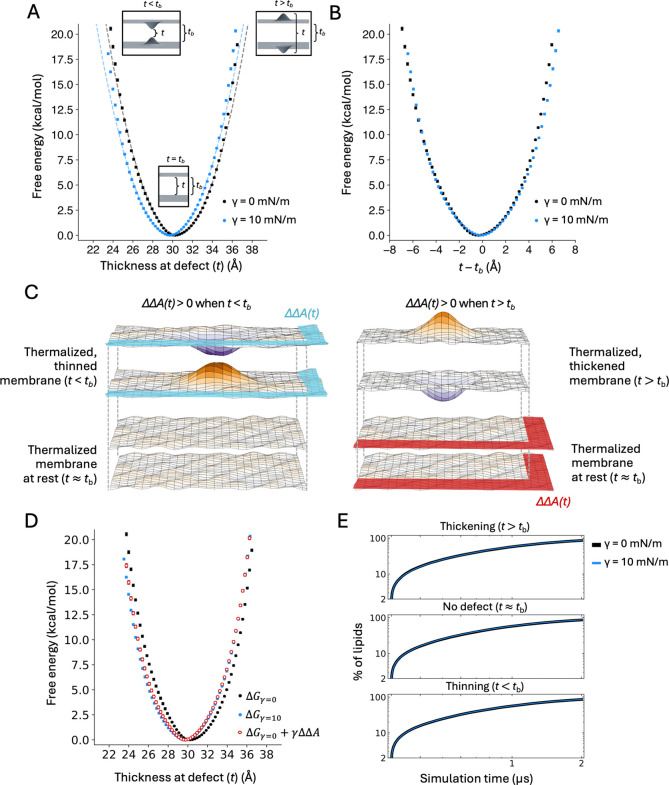
Effect of lateral stretch on the energetics of thickness perturbations. (**A**) Potential of mean force (PMF) for a localized membrane thickness defect. The free energy is plotted as a function of the thickness at the center of the induced defect, t, under applied lateral tension γ=10mN/m (blue) or without any tension applied (black). The PMF curves were calculated with the Multi-Map method in combination with umbrella sampling, for an 800-POPC lipid bilayer. Each umbrella-sampling window was 2 μs. Fragments of 400 ns were used to estimate average values of the PMF (circles) and standard deviations, σ (bars); the error bars in the plot (2σ) range from 0 to 0.17 kcal/mol. The dashed lines are quadratic fits i.e. *y* = *a*(*x* – *b*)^*2*^ of the PMF data below 5 kcal/mol, with *a* = 0.41 kcal/Å^2^×mol and *b* = 30.4 Å for γ = 0 mN/m and *a* = 0.38 kcal/Å^2^×mol and *b* = 29.7 Å for γ = 10 mN/m. (**B**) Same as in (A), except the PMF data is plotted against *t* – *t*_b_, where *t*_b_ is the bulk thickness of membrane. **(C)** Schematic illustrating the changes in the projected area of the membrane as a result of localized thickening or thinning, with color intensity (purple or orange) indicating larger amplitudes relative to the monolayer midplane. ΔΔA(*t*) is the difference in projected area between two thermalized states of the membrane, namely a reference, undeformed state with *t* ≈ *t*_b_ and a deformed state with *t* > *t*_b_ or *t* < *t*_b_. Note that ΔΔA(*t*) > 0 for *t* > *t*_b_, and ΔΔA(*t*) < 0 for *t* < *t*_b_. (**D**) Same as in (A), with an additional free-energy profile (red) obtained by adding a γΔΔA(*t*) term to the PMF(*t*) profile calculated with γ = 0 (black). The errors for this additional profile, ranging from 0.11 to 0.20 kcal/mol, are the sum of the errors in the PMF for γ = 0 and the errors in ΔΔA calculated with Eq. 9. (**E**) Evaluation of the impact of the thickness defect on the lipid dynamics. The plots quantify the degree of lipid mixing, which we define as the percentage of lipids *j* that reside for at least 10 ns in a 12 Å shell around each lipid *i*, for each leaflet. Full mixing is reached when this quantity is 100% i.e. all the possible lipid pairs *ij* have been observed to be first-neighbors for at least 10 ns. Note this condition is nearly achieved after 2 μs of simulation. Data is shown for three magnitudes of the thickness defect, namely *t* > *t*_b_, *t* ≈ *t*_b_, or *t* < *t*_b_, with or without applied tension γ = 10 mN/m. This comparison shows neither the thickness perturbation of the application of tension influences the lipid exchange dynamics.

The resulting PMF curves are striking in that they demonstrate that seemingly minor changes in thickness, relative to the bulk value, are energetically very costly, with or without tension (Figure 5A). For example, the cost of thinning or thickening the membrane by only 5 Å is ~10–12 kcal/mol. These results explain why spontaneous thickness fluctuations in lipid bilayers at rest are minimal. They also underscore the dominant effect that amino-acid solvation energetics can have on membrane morphology, as noted above.

Interestingly, the free-energy profiles obtained under either tension condition are roughly harmonic in shape, as we observed for global bending (Figure 5A). However, deviations from this quadratic form are increasingly apparent as the defect becomes more pronounced (Figure 5A). An asymmetry in the PMF profiles also becomes evident at these more extreme values, reflecting the fact that membrane thinning and thickening are not structurally equivalent processes.

Because the bulk thickness value $t_{b}$ decreases under stretch (Figure 2A), the PMF obtained when the membrane is tensioned with γ=10mN/m is shifted to the left of that obtained without applied tension (Figure 5A). Specifically, for the POPC membrane used in this study, this shift is ~0.7 Å. The implication is that features of a protein surface that require a certain perturbation in membrane thickness might be more or less attainable under stretch than otherwise. For example, for the POPC bilayer examined here, a perturbation whose hydrophobic thickness is 25 Å would be ~3 kcal/mol less costly, or 100 times more feasible, under γ=10mN/m than in a tensionless state, while the opposite would be expected for a perturbation whose thickness is 35 Å.

Aligning the free-energy curves calculated with and without tension by their respective minima (Figure 5B) reveals that the cost of thinning the membrane relative to the bulk ($t<t_{b}$) is virtually independent of tension up to about 6 Å, but the curves diverge beyond that point. By contrast, thickening of the membrane relative to the bulk ($t>t_{b}$) is generally less costly when the membrane is stretched. Based on our findings for the global bending deformations and for the thermal fluctuations at rest, we reasoned that this differential effect might again stem from changes in membrane area induced by the thickness perturbation, and how those changes are penalized or favored under stretch. To examine this hypothesis, we deduced the relative change in projected area caused by this perturbation, ΔΔA, from an evaluation of t, the thickness at the center of the defect. More specifically, we used:

(9) $$\Delta \Delta A\left(t\right)=\frac{4\pi \sigma^{2}\left(t-t_{b}\right)}{t_{b}}$$

where σ=8Å is the half-width of the two-dimensional Gaussian function used to create the thickness defect, $2\pi \sigma^{2}$ is the integral of that function, and the additional factor of 2 accounts for the fact both bilayer leaflets are simultaneously perturbed.) As for the bending deformation, we evaluated the mean value of t for each of the umbrella-sampling simulations used to generate the thickness defect without applied tension, and then calculated the additional free-energy term γΔΔA, with γ=10mN/m. Note that in this case ΔΔA is positive for the thickened defect ($t>t_{b}$) but is negative for the thinned defect ($t<t_{b}$) (Figure 5C). With γ>0, the free-energy term γΔΔA therefore penalizes the former but favors the latter.

As shown in Figure 5D, when that γΔΔA term is added to the PMF calculated for the tensionless condition, the resulting curve is in excellent agreement with the PMF calculated under γ=10mN/m. Thus, also for localized perturbations in thickness we find that lateral stretch alters the energetics of membrane remodeling, and that this free-energy differential can be accurately deduced from the change in projected membrane area (ΔΔA) caused by the deformation. Finally, it is worth clarifying that neither tension nor the membrane thickness defects generated in this analysis have a discernable impact on the lipid dynamics, when compared to an undeformed state (Figure 5E). The resistance to thinning or thickening is therefore structural in nature.

### Does applied lateral stretch influence the dynamics of lipids in the membrane?

The results presented above demonstrate that lateral stretch influences the average structure of the membrane, the magnitude of its shape fluctuations at rest, and the energetics of morphological changes that might be induced extrinsically, for example by proteins. However, lateral stretch is often portrayed as an effect that exerts through-space forces on the lipid molecules within the membrane, running parallel to the direction of stretch and influencing their dynamics at the molecular level (64–66). In this view, forces resulting from the application of lateral tension can drive structural changes in proteins, or foster the dissociation of lipid molecules from binding sites on their surface.

To conclude our analysis of the effects of lateral tension on lipid bilayers, we sought to test this notion. To do so, we used the Multi-Map method to induce the enrichment or depletion of a specific subset of lipid molecules in a certain volume within the membrane, with and without applied tension, evaluating in each condition the rate at which those lipids enter (and exit) the target volume. We also quantified the free-energy cost of this process of enrichment or depletion, and compared the result with what would be mathematically expected for a homogeneous membrane at rest. For simplicity, we used a cylinder of 10-Å radius as the target volume, which approximately corresponds to 8 lipids per leaflet (both with and without tension). The subset of lipids that were subject to enrichment or depletion comprise 50% of the membrane. These “special” lipid molecules, referred to as ${POPC}_{\text{special}}$, were selected at random and are, of course, identical to the other lipid molecules. Therefore, any enrichment or depletion of ${POPC}_{\text{special}}$ in the target volume leaves the structure and energy of the membrane unchanged, and the corresponding gains or losses in free energy would consist entirely of entropy.

**Figure 6.**
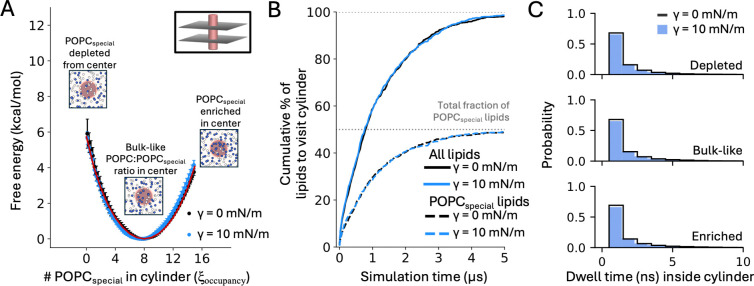
Applied lateral tension does not drive lipid dynamics. (**A**) Potential of mean force (PMF) for enriching or depleting lipids designated as ${POPC}_{\text{special}}$ in a cylindrical volume of 10-Å radius at the center of the membrane, under no stretch (black) or under applied lateral tension of 10 mN/m (blue). The free energy is plotted as a function of the number of ${POPC}_{\text{special}}$ lipids in the target area, $\xi_{\text{occupancy}}$. The PMF data were calculated with the Multi-Map method in combination with umbrella sampling, for an 800-POPC lipid bilayer. Inset figures capture a snapshot of the simulation system in the umbrella-sampling windows calculated for $\xi_{\text{occupancy}}$ values of 0, 7, and 15. Each umbrella-sampling window was 5 μs. Fragments of 1 μs were used to estimate average values of the PMF (circles) and standard deviations, σ (bars); the error bars in the plot (2σ) range from 0.01 to 0.82 kcal/mol. The red line shows the PMF curve that is mathematically predicted purely based on entropic factors, using Eq. 10. (**B**) Cumulative percentage of lipids in each leaflet of the membrane that enter the target cylindrical volume after a certain simulation time, with (blue) or without applied tension (black), counting all lipids (solid lines) or only ${POPC}_{\text{special}}$ lipids (dotted lines). These data derive from the umbrella-sampling window for $\xi_{\text{occupancy}}$ = 7; analogous results were obtained for other values of $\xi_{\text{occupancy}}$ (Figure S5). (**C**) Distribution of observed dwell-times of ${POPC}_{\text{special}}$ lipids inside the cylinder, in each of the umbrella-sampling windows depicted in (A). Data obtained with applied tension (blue, filled) and without (black, unfilled) are compared. Bin widths for the two tension conditions differ for clarity of visualization.

As shown in Figure 6A, the Multi-Map simulations successfully sampled all possible occupancy states of the target volume, ranging from one in which no ${POPC}_{\text{special}}$ lipids are found in the target volume (in which case only “normal” POPC lipids occupy the volume) to another in which the only lipids residing in the volume are ${POPC}_{\text{special}}$ (see also Figure S5A). Logically, not all these states are equally probable; both with and without lateral stretch, the PMF curves reflecting the free-energy cost of this enrichment or depletion show that the most probable states are those in which 50% of the lipids in the target volume are “special” lipids, while “normal” lipids fill the rest. This is the expected result for a condition that preserves the natural dynamics of the membrane, since as mentioned, ${POPC}_{\text{special}}$ comprises 50% of the lipids in the membrane. Indeed, not only the minima but the totality of the PMF curves derived from the Multi-Map simulations reproduce what would be mathematically predicted for a volume roughly equivalent to 8 lipids per leaflet, given that the membrane comprises 800 POPC lipids, 50% of which are designated as ${POPC}_{\text{special}}$ (Figure 6A). That is:

(10) $$G(n)=-k_{B}T\;ln\;\Omega (n)=-k_{B}T\;ln\left[\frac{\frac{16!}{n!\;(16\;-\;n)!}\times \frac{(800\;-\;16)!}{(400\;-\;n)!\;(400\;-\;(16\;-\;n))!}}{\frac{16!}{8!\;(16\;-\;8)!}\times \frac{(800\;-\;16)!}{(400\;-\;8)!\;(400\;-\;(16\;-\;8))!}}\right]$$

where n is the number of ${POPC}_{\text{special}}$ lipids that reside in the target volume.

We next quantified whether mechanical tension influences the rate at which lipids of either type enter (and exit) the target volume. As demonstrated in Figures 6B and 6C, we detected no discernible effect on lipid dynamics, even for a sizable tension value of 10 mN/m. That is, the rate at which lipids of either kind entered the target volume, thereby replacing other lipids therein, was unchanged when the membrane was laterally stretched (see also Figure S5B). In both conditions, we observe that after 5 μs of simulation virtually every single lipid molecule in the bilayer, whether designated as ${POPC}_{\text{special}}$ or not, has entered the target volume at least once, and dwelled therein for some time before exiting. As expected for Brownian motion, these single-molecule dwell times vary widely, from tens of nanoseconds to microseconds; yet, these distributions are also largely unchanged under lateral stretch (Figure 6C). In conclusion, we detect no evidence of a through-space lateral force that influences the dynamics of lipids when the membrane is stretched. As demonstrated in the previous sections, lateral tension does influence the structure of the membrane and its conformational energetics, but its only effect on lipid dynamics is a minor increase in overall diffusivity.

### Localized thinning and thickening defects in a mixed membrane

As mentioned above, application of lateral tension diminishes the bulk thickness of the membrane, $t_{b}$; for POPC, in our simulation conditions, that shift is about 0.07 Å per mN/m of tension (Figure 2). Membrane thinning, however, can also result from changes in lipid composition. In cells, such changes occur in response to variations in temperature, nutrient availability, or stimuli (67). We reasoned, therefore, that shape perturbations in a homogeneous membrane under tension might be energetically comparable to those in a membrane whose altered lipid composition leads to a smaller $t_{b}$.

To examine this hypothesis, we calculated the energetic cost of the localized thickness defect studied above for a tensionless POPC bilayer after addition of DLPC lipids (di12:0 PC), in a 1:1 ratio. Because DLPC features shorter alkyl chains, a 1:1 POPC:DLPC membrane is expected to be thinner, on average, than a pure POPC bilayer. Indeed, we observe that the bulk-membrane thickness of the mixed bilayer is about 2.3 Å smaller. The reduction in $t_{b}$ caused by the addition of DLPC to a POPC membrane, in equal amounts, is therefore considerably larger than that caused by a lateral tension of 10 mN/m (Figure 2).

**Figure 7.**
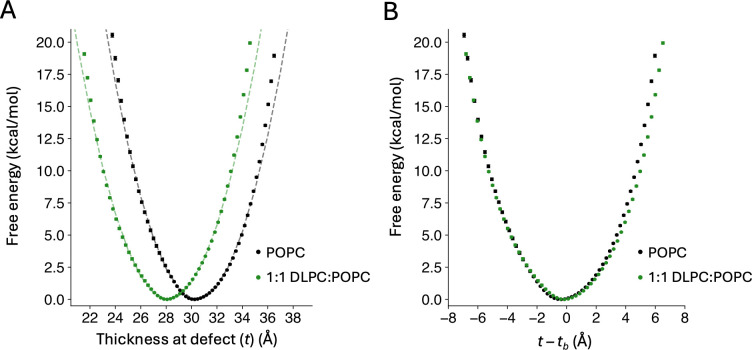
Influence of lipid composition on the energetics of thickness perturbations. (**A**) Potential of mean force (PMF) for a localized membrane thickness defect. The free energy is plotted as a function of the hydrophobic thickness at the center of the induced defect, *t*, in POPC (black) or 1:1 POPC:DLPC (green) membranes. The PMF curves were calculated with the Multi-Map method in combination with umbrella sampling, for an 800-lipid bilayer with the indicated composition. Each umbrella-sampling window was 2 μs for the POPC membrane and 7 μs for the mixed membrane. Fragments of 400 ns (POPC) or 1.4 μs (1:1 POPC:DLPC) were used to estimate average values of the PMF (circles) and standard deviations, σ (bars); the error bars in the plot (2σ) range from 0 to 0.15 kcal/mol. The dashed lines are quadratic fits i.e. *y* = *a* (*x* – *b*)^*2*^ of the PMF data below 5 kcal/mol, with *a* = 0.41 kcal/Å^2^×mol and *b* = 30.4 Å for POPC, and *a* = 0.39 kcal/Å^2^×mol and *b* = 28.1 Å for 1:1 POPC:DLPC. (**B**) Same as in (A), except the PMF data are plotted against *t* – *t*_b_, where *t*_b_ is the bulk thickness of membrane.

Figure 7A compares the PMF profiles for creating a localized thickness defect in pure POPC and in the 1:1 POPC:DLPC bilayer, without any tension applied. As we observed when contrasting pure POPC with and without applied tension, the PMF curve for the membrane with a smaller $t_{b}$ shifts to the left. The magnitude of the shift caused by the addition of DLPC is however larger than that caused by lateral stretch, consistent with the larger change in $t_{b}$. As a result of this shift, the range of hydrophobic thicknesses that are realistically attainable are very different for the mixed bilayer than for pure POPC. For example, a defect that reduces the hydrophobic thickness to 25 Å is ~7 kcal/mol less costly in 1:1 POPC:DLPC than in pure POPC (or about 100,000 times more feasible); conversely, a defect that increases the hydrophobic thickness to 33 Å is ~7 kcal/mol more costly in the mixed membrane.

**Figure 8.**
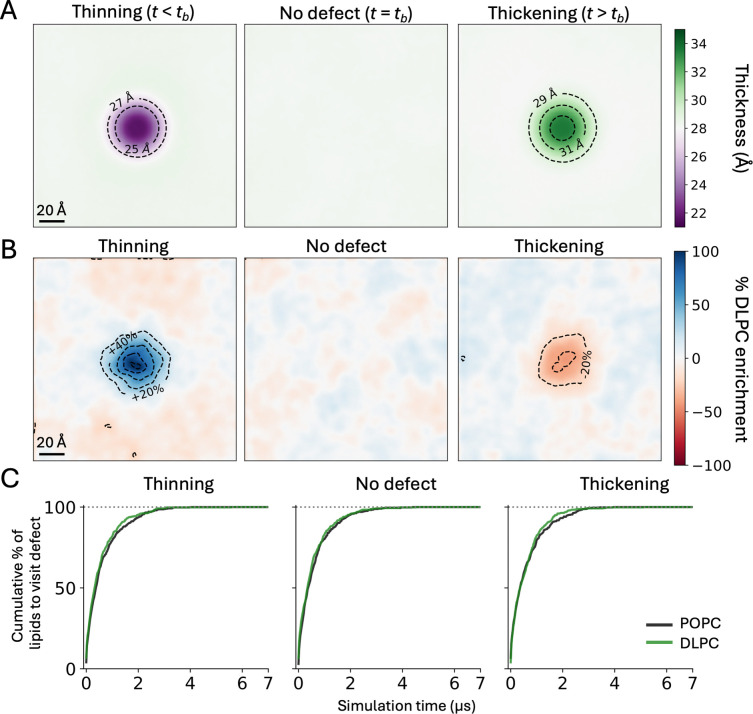
Spatial redistribution of POPC and DLPC lipids induced by membrane thickness defects. (**A**) Two-dimensional map of the hydrophobic thickness of the membrane for three different magnitudes of the induce central defect, namely *t* < *t*_b_ (left), *t* ≈ *t*_b_ (center) or *t* > *t*_b_ (right). Both POPC and DLPC lipid molecules contribute to define these maps. Dotted contour lines indicate 2-Å increments or decrements. The thickness value in the bulk, *t*_b_, is 28.2 Å. (**B**) Distribution of DLPC lipids in the three states represented in (A). An enrichment level of 0% corresponds to the bulk 1:1 POPC:DLPC ratio. Positive enrichment values reflect accumulation of DLPC relative to that bulk ratio, while negative values indicate depletion. Dotted contour lines indicate 20% increments or decrements. Note that trajectories of up to 7 μs were required for statistical convergence of these lipid-distribution maps (Figure S6). (**C**) Cumulative percentage of POPC or DLPC lipids that enter the target cylindrical volume, in each leaflet of the membrane, after a certain simulation time, for each of the states represented in (A) and (B). Note that after 4 μs of simulation all lipids have visited the target volume, and that the turnover of DLPC lipids is slightly faster.

Interestingly, overlay of the PMF curves calculated for the mixed and pure membranes shows minimal differences (Figure 7B). Addition of DLPC therefore appears not to cause significant changes in the mechanical properties of the membrane, compared with pure POPC. It is however worth noting that the spatial distribution of POPC and DLPC lipids is significantly changed by the introduction of the thickness perturbation. Specifically, in the thinned defect we observe a pronounced enrichment of the shorter-chain DLPC lipids, where the lipid content deviates from the bulk 1:1 ratio by as much as 80% (Figure 8A, 8B). Conversely, in the defect that thickens the membrane, DLPC lipids are depleted and POPC enriched (Figure 8A, 8B). These effects are entirely spontaneous, but not equivalent: DLPC enrichment in the thinned defect is stronger than its depletion in thickened defect. The likely explanation is that the presence of POPC lipids in the thinned defect is strongly penalized because it causes steric clashes between opposing leaflets; by contrast, DLPC seems to be merely disfavored in the thickened defect, as it leads to a suboptimal degree of chain interdigitation across the midplane. At any rate, it is plausible to infer that adaptations in the spatial distribution of the two lipid species in response to the changes in membrane morphology contribute to explaining why the PMF curves for pure POPC and the 1:1 POPC:DLPC mixture are shaped so similarly.

Finally, it is worth emphasizing that the observed accumulation of POPC or DLPC in the induced thickness defect does not imply that individual lipid molecules are localized, or that the lipid dynamics is somehow arrested. In fact, we observed that after 4 μs of simulation time every single lipid molecule in the membrane had visited the thickness deformation at least once, for both the thinned and thickened defects (Figure 8C). As expected, DLPC diffuses slightly more quickly than POPC, generally (Figure 8C). However, the single-molecule dwell times inside the defect were nearly identical for the two lipid types (Figure S6), indicating that enrichment or depletion of a certain species at the defect reflects differential probabilities for entry into and exit from that locale, rather than a slow-down of the lipid dynamics. From a methodological standpoint, we would argue that these interdependent observations underscore one of the key advantages of the Multi-Map simulation framework. That is, through this approach it is possible to quantitatively evaluate how variations in lipid content influence the energetics of membrane morphology, while simultaneously assessing how changes in morphology influence the spatial distribution of different lipid types.

## Discussion

Many membrane proteins exist in an equilibrium between distinct conformational states, or between different states of supramolecular organization. We tend to rationalize these phenomena solely in terms of the internal structure of these proteins, modulated perhaps by extrinsic inputs such as ligands, interacting proteins, or electric fields. It is however likely that the morphological energetics of the lipid bilayer is the environmental factor that is most influential for membrane protein structure and function, individually or in complexes. This influence has been however difficult to quantify, both experimentally and computationally, as protein structure or activity can hardly be evaluated without a membrane, or at least a medium that mimics it. Nonetheless, it is self-evident that the membrane is not a passive, infinitely adaptable medium; it features its own conformational preferences, both in terms of shape and lipid distribution. Integral membrane proteins must therefore contend with the lipid bilayer energetics; if a protein features alternate structural states that reshape the membrane or redistribute its lipid content differently, as has been demonstrated for a range of channels, transporters, and enzymes, the activity of that protein will be necessarily sensitive to extrinsic factors that alter the morphology or chemical composition of the membrane.

Mechanosensitive proteins are the most salient example of this interdependence. Well-studied ion channels like PIEZO, MscL, MscS, and their homologs belong to this class of proteins. Like other ion channels, these proteins naturally exist in an equilibrium between conducting (open) and non-conducting (closed) states. What differentiates mechanosensitive channels is that this equilibrium shifts in response to applied lateral stretch, even in the absence of any other proteins or cellular components. Despite decades of study, however, the precise role of the membrane in this form of mechanotransduction remains unclear. A clear understanding of lateral tension as a physical input has apparently been also lacking. As we have shown, it is a misconception that applied tension produces through-space forces that can drive the dynamics of lipids in the membrane or cause structural movements in proteins. This notion is perhaps inspired by the influence of electric fields on the motion of charged species or structural elements within them; at any rate, based on our studies of structure and dynamics of membrane under stretch, we see no physical basis for such an interpretation.

How, then, does applied lateral stretch affect the membrane, and the proteins therein? To examine this question, we leveraged a recently developed molecular-simulation method, known as Multi-Map, to quantify the energetics of different forms of membrane perturbation with and without applied tension. The results reveal that applied tension influences the energetics of these perturbations differently. Global bending deformations that otherwise preserve the volume of the membrane become more costly under stretch, as do localized deformations that cause the membrane to thicken. By contrast, membrane thinning becomes easier under stretch. Interestingly, the reason for these effects is not that tension influences the intrinsic mechanical properties of membrane, such as its bending modulus. Instead, the effect of lateral stretch is to penalize deformations that cause the projected area of the membrane to decrease, while those that expand that area are favored. This conclusion does not rest on any pre-conceived model or theory: the free-energy profiles presented in this study, and the physical descriptors used to interpret them, stem solely from the intermolecular forces and preferred configurations observed during our MD simulations.

It is also interesting that the concept of projected area change also explains the effect of tension on membranes at rest. In this resting state, thermalization will cause a membrane to show a spectrum of fluctuations in shape. Our analysis demonstrates how the application of tension selectively suppresses the fluctuation modes with a larger amplitude and wavelength, precisely because these modes cause the projected area of the membrane to deviate from the ideal ground state. Again, this observation stems solely from the simulated trajectories, though in this case the simulation data confirms what existing analytical theories had predicted.

Does this perspective provide insights into the origins of mechanosensation? While more work will be required to answer this question conclusively, we believe our analysis offers some important clues. The closed state of PIEZO induces a dome-shaped deformation in the membrane, 200–300 Å in length, which is largely eliminated when the protein transitions to the open state (68–72). For the reasons stated above, lateral stretch will make the large deformation required by closed-state PIEZO very costly, even at modest tension values. And indeed, PIEZO is known to gate at tension values as low as 1–2 mN/m (73,74). MscS also induces clear perturbations of the membrane thickness and curvature in the closed state, of the type that would be increasingly disfavored by tension. The extent and magnitude of these perturbations are however much more limited than those observed for PIEZO, implying that the energetic penalty associated with these deformations becomes dominant only for larger values of the applied tension. And indeed, MscS gates at more moderate tensions, in the 5–6 mN/m range (2,75–77). Finally, it is worth noting that while MscL does not cause pronounced localized defects in the surrounding membrane, such as those observed for MscS, it does feature a slightly smaller hydrophobic thickness in its open state, compared to its closed state. Based on our examination of the energetics of thickness defects, we would anticipate that the closed state of MscS is disfavored under stretch, albeit for only for large tension values. Consistent with this theory, MscL gates at 10–12 mN/m (78,79).

This perspective of membrane energetics also provides a satisfactory explanation to a related empirical observation, namely that reconstitution of a protein in lipid environments featuring different hydrophobic thicknesses can produce different structures, if that protein deforms the membrane differently in alternate conformations. The structure of MscS, for example, was repeatedly resolved in the closed state, i.e. the preferred state under no tension, in conditions where the protein was solubilized in detergent or long-chain lipids or detergents (65,76,77,80). Ultimately, solubilization in nanodiscs of short-chain lipids (2,77) revealed the open state that is favored under lateral stretch, even though no tension was applied in the experiment. Our simulations clearly show that the reason for this observation is that short lipids cause the energetics of membrane deformations to shift similarly to lateral stretch. The perturbations induced by MscS in the closed state, which as mentioned cause the membrane to thicken, are just as unfavorable under tension as they are when the membrane is thinner overall.

In conclusion, we have demonstrated that lateral stretch alters the morphological energetics of lipid bilayers, supporting the notion that deformations in membrane curvature and thickness underlie the controlling effect of applied tension on the activity of mechanosensitive ion channels (2,68–72). We have also shown that tension has a marginal effect on lipid dynamics, indicating that it does not directly influence the lifetime of specific protein-lipid interactions that might occur. Clearly, much more ground remains to be covered to attain a comprehensive understanding of mechanosensation, and more broadly, of the growing number of biological processes known to entail membrane deformations of different magnitude and scale (1,2,8,13,81–84). We anticipate that molecular-simulation methods, such as Multi-Map (37), will continue to reveal important insights in this area of research, provided these methods are designed to enable realistic modeling and quantitative evaluation of non-native bilayer states, both in terms of shape and lipid distribution.

## Supplementary Material

Supplement 1

## Data Availability

Simulation inputs and representative outputs are available upon request to the authors. Code developed specifically for this study can be freely downloaded as noted above.

## Statement of Significance

Cells have evolved the ability to sense mechanical forces, such as pressure or stretch, through specialized proteins embedded in their membranes. How exactly the membrane transduces these stimuli to the proteins therein has been unclear. Using state-of-the-art computer simulations, we show that stretching a membrane does not result in forces that pull or push on the individual lipid molecules that constitute the membrane. Instead, lateral tension alters the energetics of reshaping the membrane. This shift in plasticity explains why several well-known force-sensing proteins switch between active and inactive states at specific tension values observed experimentally. We also show that altering the lipid composition of the membrane can produce the same effect as lateral stretch, without any applied force.
